# First Report of Intestinal Myiasis Due To *Eristalis tenax* in Iran

**Published:** 2010-06

**Authors:** MR Youssefi, SAA Sefidgar, M Abouhosseini Tabari

**Affiliations:** 1 Department of Veterinary Parasitology, Islamic Azad University, Babol - Branch, Iran; 2 Department of Parasitology and Mycology, Faculty of Medicine, University of Babol, Iran; 3 Department of Veterinary Pharmacology, Faculty of Veterinary Medicine, University of Tehran, Iran

**Keywords:** *Eristalis tenax*, Intestinal myiasis, Rat tail maggot

## Abstract

*Eristalis tenax*, belonging to order Diptera, family Syrphidae seldomly causes intestinal myiasis. Intestinal myiasis caused by *E. tenax* larvae is a rare manifestation found in both humans and other vertebrate animals. We report a 22-year-old woman presented with this myiasis. The larva in her stool sample was identified as *E. tenax* related to its typical morphology and authentic clues. Lack of specific control measures in the domestic water supply system was the most probable cause of this infestation.

## Introduction

Infestation of live humans or vertebrate animals with larvae of *Diptera* (fly) species is known as myiasis for part of their life cycle, the larva feed on dead or living tissue or the ingested food of the host. Pseudomyiasis is the term used for deposition of maggots on faeces immediately after they are passed. Recognition of dead larva in stool also comes into this category, as host infestation has not been established (1). A few patients with intestinal myiasis with drone fly larvae have been described previously, with the mode of infestation presumed to be consumption of water or food contaminated with fly larva or eggs.

The rat-tailed maggot usually breeds in drains, sewage pools, and other stagnant water. Although the larva lives on decaying organic matter, they must breathe air (2). Doubts have been expressed about the theory that accidentally ingested fly larva could survive in the environment of the gastrointestinal tract. Zumpt proposed an alternative kind of intestinal myiasis due to *Eristalis tenax* called "rectal myiasis". Flies, attracted to faeces, may deposit their eggs or larva near or into the anus, and the larva then penetrate further into the rectum (3).

In the present study, we describe the detection of *E. tenax* larva in feces, which is the first report of an infected human in Iran.

## Case Report

**Fig. 1:**
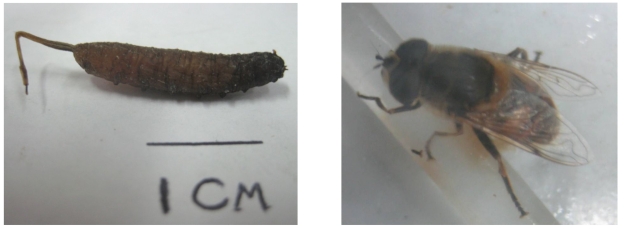
The specimen, apparently passed with stool, was identified as a "rat-tailed maggot", or larva of fly *Eristalis tenax* (left) and adult fly (right) (Photographed by MR Youssefi)

A 22-year-old woman living in a rural part of Babol, Mazandaran (North of Iran) represented a history of excreting active swimming "fish-like creatures" in her stool. There were no other symptoms such as anal purities (apart from a sense of revulsion). Specimens obtained from the patient were cylindrical larva measured about 2.5±0.5 cm long with a "tail". Based on the morphology characteristic according to Hartley (7) they were identified as "rat-tailed maggots”, or larva of the introduced drone fly (*E. tenax*, Fig. 1). The patient excreted two or three to five larva daily over a 2–3 weeks period. She remained asymptomatic. There was nothing to suggest except the patient had a low standard of hygiene.

Some of larva excreted by the patient kept in plates in laboratory, after 2 weeks changed to adult flies (Fig. 1). Adult flies were used to confirm the identification. Pidrolax was used as an empirical treatment.

## Discussion

Gastrointestinal myiasis caused by the cosmopolitan drone fly *E. tenax* is classified as pseudomyiasis, given the biology of the fly and that it occurs in an accidental manner. Its presence, in the surface of digestive tract is responsible for the pathologic physiology, in general of lesser severity than that caused by the obligatory or facultative parasites. Kun et al. reported that larva obtained from faeces of two patients was identified as *E. tenax* (4). In other study, Duboies et al. reported an unusual case of indigenous intestinal myiasis caused by *E. tenax* during summer 2003 in a 36-year-old man living in Belgium (5), the patient complained of diarrhea and intestinal rumbles. Mumcuoqlu et al. reported a 58 years old woman presented with painful mixing and bilateral costo-lumbar pain. The larva in her urine sample was identified as *E. tenax* related to its typical morphology (6).

Intestinal myiasis in humans is probably an accidental myiasis related to ingestion of contaminated uncooked food or water containing fly larvae. Most larvae are destroyed by the digestive juice, but others are able to live in the intestinal tract and produce intestinal distress. Moreover, the larvae can also exceptionally reach the intestinal tube through the anus (rectal myiasis). In urban areas of developed countries cases of intestinal myiasis are rare, most have occurred in countries where nutritional and sanitary conditions are unsatisfactory. Intestinal myiasis due to larvae of the drone fly *E. tenax* is reported sporadically from various countries and is briefly mentioned in major textbooks of tropical medicine and parasitology, but to date no case was reported in Iran.
